# Extracellular nicotinamide phosphoribosyltransferase visfatin activates JAK2-STAT3 pathway in cancer-associated fibroblasts to promote colorectal cancer metastasis

**DOI:** 10.1007/s13258-024-01596-6

**Published:** 2024-12-06

**Authors:** Yun Lei, Dan Shu, Jianyu Xia, Tao Zhang, He Wei

**Affiliations:** 1 School of Bioscience and Technology, Chengdu Medical College, Chengdu, China; 2 School of Basic Medical Science, Chengdu Medical College, Chengdu, China; 3 Pathological Diagnosis Center, Zhoushan Hospital of Zhejiang Province, Zhejiang, China; 4 The Second Affiliated Hospital of Chengdu Medical College, China National Nuclear Corporation 416 Hospital, Chengdu, China

**Keywords:** Colorectal cancer, Cancer-associated fibroblasts, Visfatin, Metabolism, Cancer therapy

## Abstract

**Background:**

Metastasis is one of the major challenges in the treatment of colorectal cancer (CRC), during which cancer-associated fibroblasts (CAFs) in the tumor microenvironment are critically involved.

**Objective:**

In this study, we aim to explore the regulatory role of extracellular nicotinamide phosphoribosyltransferase Visfatin and its impact on CRC metastasis.

**Methods:**

To examine the effect of visfatin on CAFs, human CRC tissue-derived CAFs were exposed to visfatin, and the expression of inflammatory factors, activation of JAK-STAT pathway and production of ROS in CAFs were assessed. To examine the effect of visfatin-treated CAFs on CRC metastasis, human CRC cell line SW480 or SW620 were cultured with the conditioned medium derived from visfatin-treated CAFs, and the invasion and migration ability of SW480 or SW620 cells were evaluated by transwell migration and matrigel invasion assays.

**Results:**

Our previous study found that visfatin, a secreted form of nicotinamide phosphoribosyltransferase that governs the rate-limiting step of NAD synthesis, promoted CRC metastasis. However, little is known about the effect of visfatin on CAFs. The conditioned medium derived from visfatin- treated CAFs promotes the migratory and invasive capability of CRC cells, and enhance lung metastasis in mouse model. Visfatin treatment stimulated the expression of a couple of inflammatory factors in CAFs, which was mediated by visfatin-induced activation of JAK- STAT pathway and accumulation of ROS. Inhibition of JAK-STAT pathway or neutralization of cellular ROS attenuated visfatin-mediated migration and invasion of CRC cells.

**Conclusions:**

The present work highlights a critical role of visfatin in the crosstalk between CRC cells and CAFs, which moonlight as a non-metabolic extracellular signal molecule to hijacks JAK-STAT pathway in CAFs to promote CRC metastasis.

**Supplementary Information:**

The online version contains supplementary material available at 10.1007/s13258-024-01596-6.

## Introduction

Colorectal cancer (CRC) is a common gastrointestinal cancer with high incidence and high mortality rates (Sung et al. 2021). Metastasis is responsible for ~ 90% of cancer-related death (Steeg 2006), and around 50% of CRC patients will develop distant metastases (Stewart et al. 2018). The tumor microenvironment, a complex environment for tumor cells to survive, is one of the necessary conditions for CRC metastasis (Quail and Joyce 2013). The tumor microenvironment is composed of various types of stromal cells and extracellular matrix, which interact with tumor cells to promote tumor survival, progression and metastasis (Zhan et al. 2023). Therefore, targeting the homeostasis of tumor microenvironment will provide a promising strategy for CRC therapy.

Cancer-associated fibroblasts (CAFs) are pivotal stromal cells of the tumor microenvironment and plays versatility and sophisticated roles in regulating tumor growth and metastasis (Zhang et al. 2023). CAFs are known to regulate both itself and neighboring tumor cells to promote tumor metastasis by secreting inflammatory cytokines, growth factors, and components of the extracellular matrix (Chen and Song 2019). However, CAFs are a highly heterogeneous group of cells. The tumor-promoting CAFs must be activated to exert their tumor-promoting functions. Previous studies have shown that secreted proteins (such as VEGF and TGF-β) in the tumor microenvironment are essential for the activation and phenotypic remodeling of CAFs, thereby promoting tumor metastasis (Nwani et al. 2016).

Visfatin, also known as eNAMPT (Extracellular Nicotinamide phosphoribosyltransferase, also called pre-B-cell colony enhancing factor), plays a central role in mammalian cell metabolism by contributing to nicotinamide adenine dinucleotide biosynthesis. NAMPT also performs diverse functions as a secretory protein, which is a novel adipokine involved in inflammation, cell proliferation, apoptosis, cellular migration, and metastasis of cancer cells (Semerena et al. 2023). Our previous study has shown that the level of visfatin is upregulated in CRC and associated with tumor stages (Yang et al. 2016). It has also been reported that visfatin activates the TGF-β signaling pathway, suggesting that visfatin may play a role in regulating the phenotypic remodeling of CAFs (Soncini et al. 2014). However, the precise role of visfatin in the regulation of CAFs is largely unknown.

In this study, we isolated CAFs from human CRC tissues and demonstrated that visfatin activates JAK2-STAT3 signaling in CAFs in a ROS dependent manner, thereby promoting the metastasis of CRC. This study provides novel insights into the regulatory role of CAFs in CRC metastasis, and will facilitate the development of novel therapeutic strategies for advanced CRC.

## Materials and methods

### Human samples

The human CRC tissues and adjacent tissues were obtained from CRC patients in the Second Affiliated Hospital of Chengdu Medical College. All experiments using these specimens were approved by the ethics committee (ChiCTR2100047942) and performed strictly abiding by relevant regulations of the Declaration of Helsinki under the prerequisite of obtaining written informed consent. A part of tissues was used to isolate CAFs and NFs (normal fibroblasts), and the other part was stored in liquid nitrogen for subsequent experiments. The ethics approval was been approved by the Ethical Committee of Chengdu Medical College (Sichuan, China) under the Helsinki Declaration.

### Mice model

All animal experiments were approved by the Animal Ethical Committee of Chengdu Medical College (Sichuan, China). SW480 cells were incubated with conditioned medium for 48 h, and then the cells were collected (2 × 10^6^ cells/mice) and injected into nude mice from the tail vein to establish the lung metastasis tumor model.

### Isolation of primary CAFs and NFs

Primary CAFs were isolated from human CRC tissues, and NFs were isolated from non-cancerous adjacent tissues. Fresh tissue was washed twice in PBS, followed by stripping unwanted tissues and fascia. The cleaned tissue was immersed in 75% ethanol for 3 min, incubated with 2% levofloxacin hydrochloride for 5 min, the tissue samples were then broken down into single-cell suspensions with sterile scissors, and the ground tissue was placed in an enzyme mixture containing collagenase and hyaluronidase and gently stirred continuously for 1–2 h under a warm bath at 37 °C. The digestive fluid was filtered using a 100 μm cell filter to remove the incomplete tissue mass. The filtered tissue was centrifuged at 1500 rpm for 10 min, and the supernatant was discarded. The precipitate was added in 5 ml of MEM medium with 20% fetal bovine serum, then mixed and incubated in an incubator with 5% CO_2_ at 37 °C. After the cells adhere to the plate, the medium was centrifuged (400 g, 4 °C, 5 min) to remove the floating cells, followed by centrifugation (3000 g, 4 °C, 20 min) to eliminate the cell debris, and CAFs or NFs were cultured with fresh medium with 20% fetal bovine serum.

### Cell culture

SW620 cells and SW480 cells were purchased from Feiouer Company (Chengdu, China) and cultured in RPMI-1640 (Biological Industries, Israel) with 10% fetal bovine serum (FBS) (Biological Industries, Israel), 1× penicillin–streptomycin solution (Biological Industries, Israel). All cells were cultured in a humidified incubator (Thermo Scientific, USA) with 5% CO2 at 37 °C.

### Preparation of conditioned medium

The NFs or CAFs used for the preparation of conditioned media were derived from the 20 samples identified in the clinical data. NFs or CAFs were incubated with fresh medium for 48 h, and the medium was collected and used as NFs conditioned medium (NFs CM) or CAFs conditioned medium (CAFs CM). For visfatin treatment, NFs or CAFs were pretreated with 200 ng/ml visfatin for indicated time in the absence or presence of 10 mM NAC or 10 µM AG490, and then replaced with fresh medium without visfatin. After incubation for 48 h, the medium was collected and used as visfatin-treated NFs CM or visfatin-treated CAFs CM.

### Western blot

The protein of cells was extracted by lysis buffer with 1% protease inhibitor cocktail (Roche, USA). Proteins were separated using 10% or 15% SDS-PAGE gel after denatured at 100 °C, then transferred to polyvinylidene fluoride (PVDF) membranes (Immobilon, China). Membranes were blocked and subsequently incubated with primary antibodies overnight at 4 °C, followed by incubating with related secondary antibodies (ZENBIO, China). The chemiluminescent HRP substrate (Millipore) was used to detect and visualize the signals of blots. The quantification was analyzed by Image J software.

### Wound healing assay

Cells were seeded in a 6-well place (Corning) and cultured overnight. In a sterile environment, a 200 μl pipette tip was used to create the vertical scratch in the cell monolayer. The cells were washed with PBS to remove cell debris. After incubation with the conditioned medium for indicated time, the width of the scratch was monitored under microscope.

### Transwell migration assay

Transwell migration assay was performed to investigate the migration ability of SW620 cells and SW480 cells. The transwell chamber (Corning) was placed in the 24 well plates, and SW620 cells and SW480 cells (1000 cells/chamber) in the transwell chamber were cultured with indicated conditioned medium for 48 h. SW620 cells and SW480 cells were then fixed with 4% paraformaldehyde for 20 min, stained with crystal violet (Sigma-Aldrich), and then washed with PBS. Cells on the top of the transwell chamber were scraped off. The migrated cells were observed by microscopy and calculated.

### Transwell invasion assay

Matrigel solution (Corning) was prepared by diluting the stock solution with sterile ice-cold deionized water. 50 μl Matrigel solution was added into the transwell chamber and incubated at 37 °C for 30 min. Cells suspended in 100 μl fresh medium or indicated conditioned medium were then added into the Matrigel coated chamber, and cultured for 72 h. Cells were then fixed with 4% paraformaldehyde for 20 min, stained with crystal violet, and then washed with PBS. Cells on the top of the transwell chamber were scraped off. The invaded cells were observed by microscopy and calculated.

#### Reverse transcription-polymerase chain reaction (RT-PCR)

Total RNA was extracted from CAFs cells using TRIzol reagent (TIANGEN, China) and dissolved in RNase-free water. After reverse transcription, the cDNA was then amplified by Taq DNA polymerase. The amplification reactions were performed as follows: one cycle of 95 °C for 10 min, followed by 40 cycles of 95 °C for 10 s, 58 °C for 30 s, and 72 °C for 15 s. The PCR products were loaded onto Ethidium Bromide-stained agarose gels for electrophoresis and the quantification of the bands was performed. All results were normalized to the ACTB mRNA levels. The PCR primers were as follows: NGF-F, ATA GCG TAA TGT CCA TGT TGT TC; NGF-R, CTT GCT CCT GTG AGT CCT GTT; BTC-F, GTA GTT TCG TTT CCT TCT GC; BTC-R, CCT GGA GGT AAC TTC ATA GC; ICAM3-F, AGG TGG ACG GCG AGT TCT TG; ICAM3-R, CTC CGT TGG TGC TCC CTG AA.

#### RNA sequencing and data analysis

Total RNA was extracted from CAFs cells using Trizol (TIANGEN, China) according to manual instruction. Subsequently, quantification of total RNA by using the Nano Drop (Thermo Scientific, USA). Each cell sample had a purity of > 90%. The RNA library construction and subsequent RNA sequencing was performed by PTMBio, China. Differential expression (DE) genes were calculated using Limma to generate differential expression tables. The upregulated and downregulated pathways were determined by KEGG analysis.

#### Inflammation cytokine array

After pretreated with 200 ng/ml visfatin for 48 h, CAFs lysate was prepared using the lysis buffer with 1% protease inhibitor cocktail (Roche, USA). The total protein concentration was determined by the BCA reagent (Thermo Scientific, USA). Cytokine array were determined using RayBiotech®® C-Series Human Cytokine Antibody Array C7 (code: AAH-CYT-7, RayBiotech, USA) as the instruction manual. Blots were analyzed using ImageLab software (Bio-Rad, USA).

#### Statistical analysis

Data are expressed as means ± SEM of three independent experiments. Statistical analysis was performed by GraphPad Prism7 software. Student’s t-test was used to analyze the significance of two group differences, and One-Way ANOVA was used for multiple comparisons. A *P*-value of *P* < 0.05 was considered significant.

## Results

### Visfatin is highly expressed in human CRC tissue-derived CAFs

**Fig. 1:**
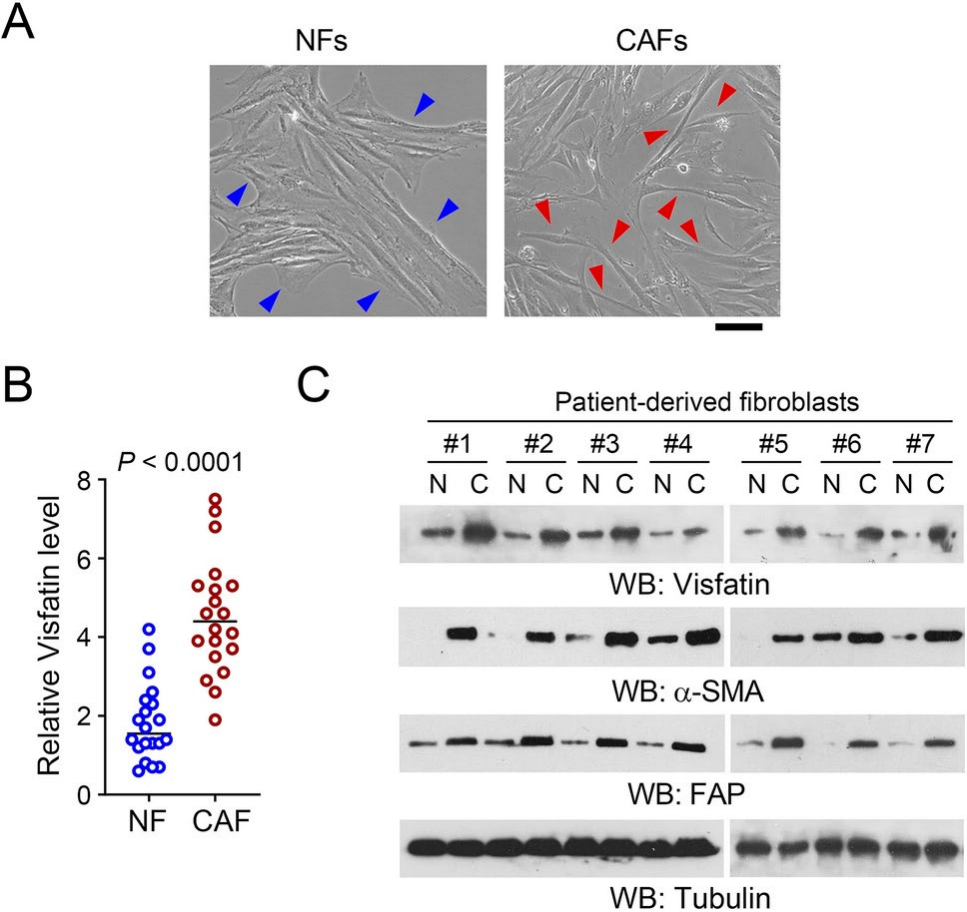
Visfatin is highly expressed in human CRC tissue-derived CAFs. **A** The morphological images of NFs and CAFs. Scale bar, 20 μm. Blue or red arrows indicated the typical morphology for NFs or CAFs, respectively. **B** Expression of visfatin in human CRC tissue-derived CAFs (n = 20) and adjacent non-cancerous tissue-derived NFs (n = 20) was analyzed by immunoblot and compared. **C** The immunoblotting with indicated antibodies were performed and representative immunoblots of seven paired CAFs and NFs were shown

To determine the expression pattern of visfatin in CAFs, we isolated CAFs and NFs from human CRC tissue or adjacent non-cancerous tissues, respectively. As shown in Fig. 1A, NFs exhibit a flat cellular morphology (indicated by blue arrows). In contrast, CAFs exhibit a long spindle shape and less cell–cell adhesion (indicated by red arrows). Immunoblot analysis of 20 paired CAFs and NFs revealed that the expression level of visfatin in CAFs is markedly higher than that in NFs (Fig. 1B and C). As expected, these isolated CAFs also showed enhanced expression of α-SMA and FAP, two CAF markers (Nurmik et al. 2020).

### Visfatin enhances CAFs-mediated CRC metastasis

**Fig. 2:**
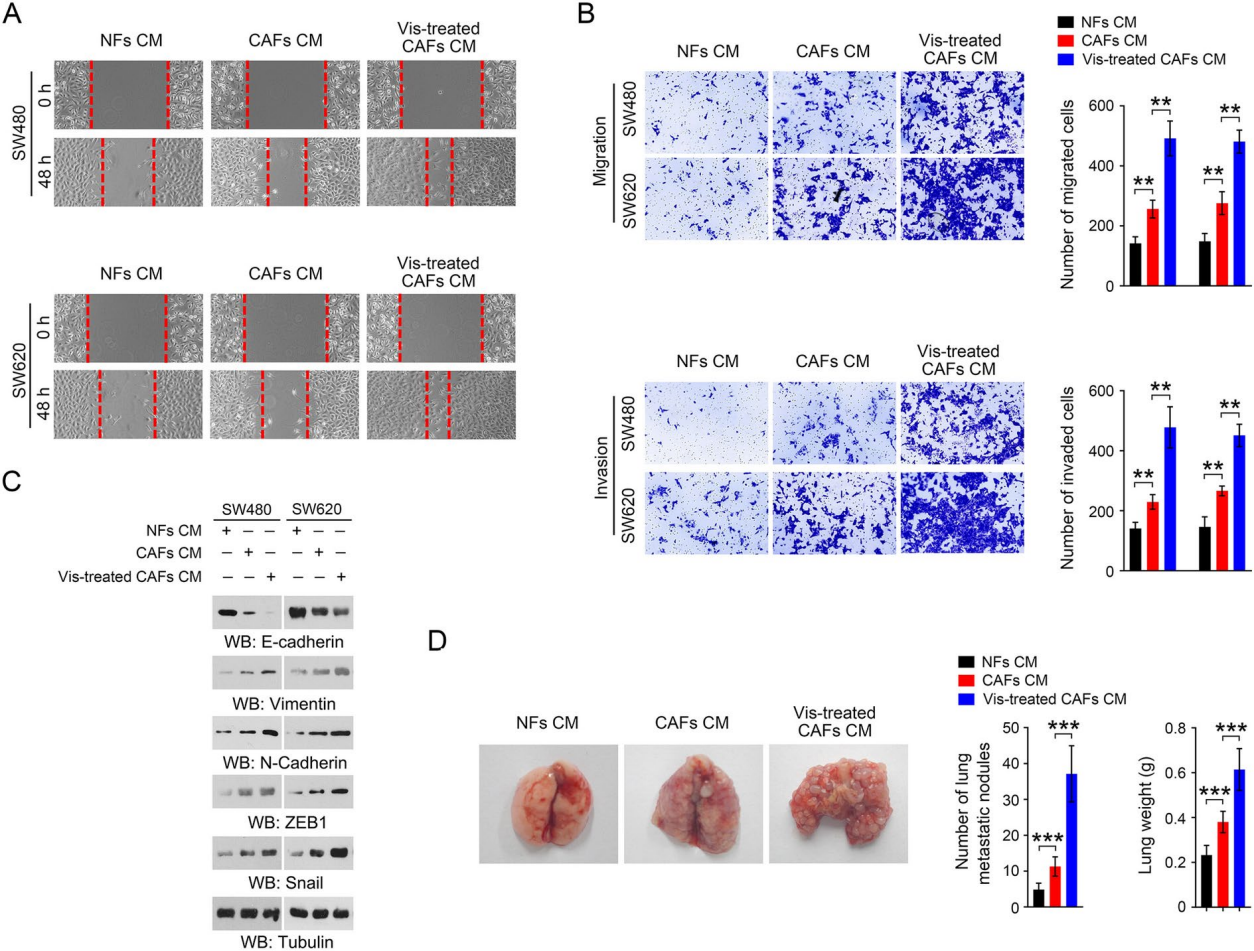
Visfatin enhances CAFs-mediated CRC metastasis. **A** The migration of SW480 and SW620 cells was analyzed by wound healing assay when these cells were incubated with the indicated conditioned medium for 48 h. **B** The migration of SW480 and SW620 cells was analyzed by transwell migration assay when these cells were incubated with the indicated conditioned medium for 48 h (the up panel). The invasion of SW480 and SW620 cells was analyzed by transwell invasion assay when these cells were incubated with the indicated conditioned medium for 48 h (the down panel). **P < 0.01. **C** The EMT related proteins were detected by immunoblotting. **D** SW480 cells were incubated with indicated conditioned medium for 48 h, and then the cells were injected into nude mice from the tail vein to establish the lung metastasis tumor model (n = 7). Representative images of mice lung were shown (left panel). The number of lung metastatic nodules and the lung weight were measured (right panel). ***P < 0.001

We previously demonstrated that visfatin promotes the metastasis of CRC cells (Yang et al. 2016). To determine whether CAFs are involved in this process, wound healing assay, transwell migration and invasion assays were performed to examine the effect of visfatin on CAFs-induced migration of CRC cells. As shown in Fig. 2A, B, incubation with visfatin-treated CAFs CM promoted the migration and invasion ability of SW480 and SW620 cells. In addition, visfatin- treated CAFs CM markedly increased the expression of epithelial-mesenchymal transition (EMT) related proteins including N-Cadherin, Vimentin, ZEB1 and Snail in SW480 and SW620 cells, companied with a decreased expression of E-Cadherin (Fig. 2C). To verify these observations in vivo, CRC cells were incubated with visfatin-treated CAFs CM for 48 h, and then injected into the tail vein of nude mice to establish a mouse lung metastasis model. Consistently, we found that, by pre-treatment with visfatin, CAFs CM enhanced the lung metastatic of CRC cells, revealed by both the increased lung metastatic nodules and lung weight (Fig. 2D). These results demonstrate that visfatin enhances CAFs-mediated CRC metastasis.

### Visfatin enhances the production of inflammatory factors in CAFs

**Fig. 3:**
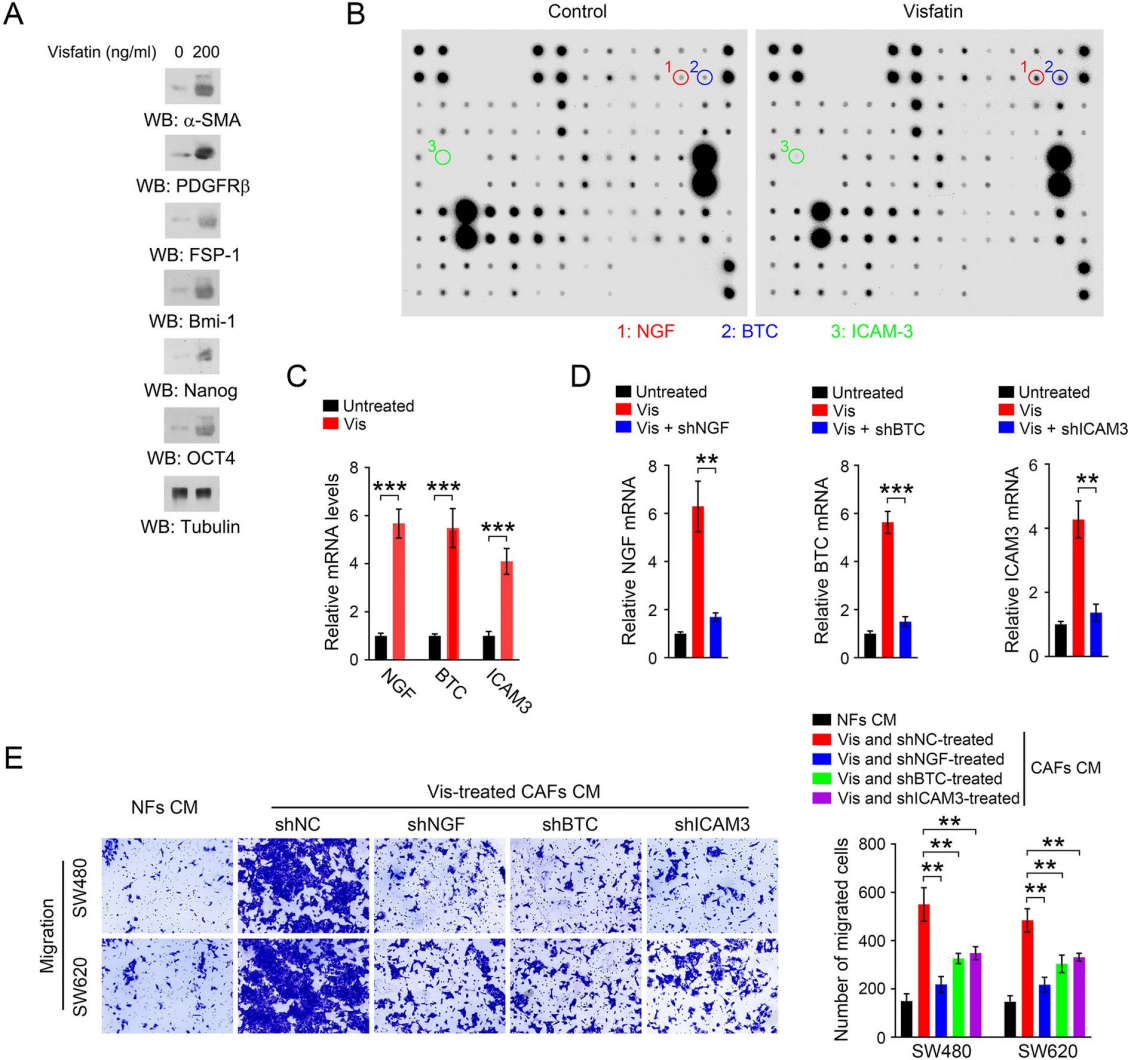
Visfatin promotes the production of inflammatory factors in CAFs. **A** CAFs were treated with Visfatin for 48 h, and immunoblot analyses were performed using the indicated antibodies. **B** CAFs were treated with Visfatin for 48 h, and inflammatory array analysis was performed. The corresponding spot of NGF, BTC or ICAM3 was annotated. **C** CAFs were treated with Visfatin for 48 h, and the mRNA levels of NGF, BTC, ICAM3 was analyzed by RT-PCR. ***P < 0.001. **D** CAFs were treated with Visfatin for 48 h with or without NGF shRNA, BTC shRNA, or ICAM3 shRNA. The mRNA levels of NGF, BTC, ICAM3 was analyzed by RT-PCR. **P < 0.01 ***P < 0.001. **E** CAFs were treated with Visfatin for 48 h with or without NGF shRNA, BTC shRNA, or ICAM3 shRNA. The CAFs were cultured with fresh medium for another 48 h, and the conditioned medium was collected. The migration of SW480 and SW620 cells was analyzed by transwell migration assay when these cells were incubated with the indicated conditioned medium for 48 h. **P < 0.01

To investigate the regulatory effect of visfatin on CAFs, we examined the expression of CAFs markers, including α-SMA, PDGFRβ, and fibroblast specific protein-1 (FSP-1), and stem cell markers, including Bmi-1, Nanog, and OCT4, in response to visfatin treatment. As shown in Fig. 3A, visfatin treatment significantly increased the protein levels of α-SMA, PDGFRβ, FSP-1, Bmi-1, Nanog, and OCT4 in CAFs. CAFs with expression of stem cell markers plays a crucial role in remodeling the tumor cell phenotype by producing various cytokines (Eisenberg et al. 2020). To identify the cytokines secreted by visfatin-stimulated CAFs which may drive CRC metastasis, the inflammatory array was performed. Compared with untreated control, visfatin-stimulated CAFs were found to produce more multiple pro-metastatic inflammatory factors, such as NGF, BTC, and ICAM3 (Fig. 3B), which was then verified by RT-PCR (Fig. 3C). This is consistent with our previous study indicating that NGF derived from tumor microenvironment is an important mediator of CRC metastasis (Lei et al. 2022). Moreover, knockdown of these inflammatory factors (Fig. 3D) markedly abolished visfatin-treated CAFs CM-induced migration of CRC cells (Fig. 3E). These findings suggest that visfatin enhances the production of inflammatory factors in CAFs, which promotes CRC metastasis.

### Visfatin activates JAK-STAT pathway in CAFs

**Fig. 4:**
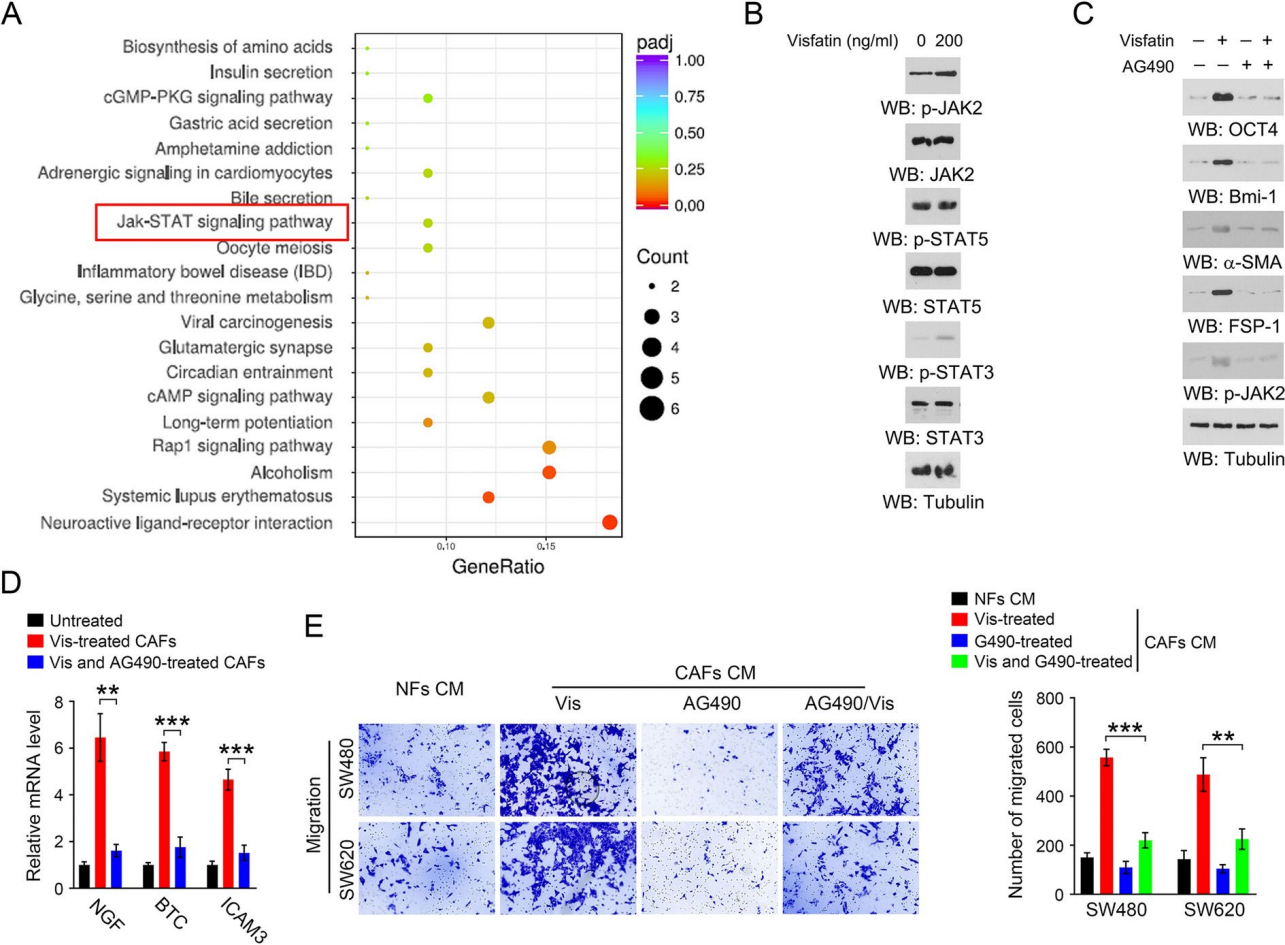
Visfatin activates JAK2-STAT3 signaling pathway in CAFs. **A** KEGG pathway enrichment analysis in CAFs under visfatin treatment. **B** CAFs were treated with Visfatin for 48 h, and immunoblot analyses were performed using the indicated antibodies. **C** CAFs were treated with Visfatin in the presence or absence of 10 µM AG490 for 48 h, and immunoblot analyses were performed using the indicated antibodies. **D** CAFs were treated with Visfatin in the presence or absence of 10 µM AG490 for 48 h. The mRNA levels of NGF, BTC, ICAM3 was analyzed by RT-PCR. **P < 0.01 ***P < 0.001. **E** CAFs were treated with Visfatin in the presence or absence of 10 µM AG490 for 48 h.The CAFs were cultured with fresh medium for another 48 h, and the conditioned medium was collected. The migration of SW480 and SW620 cells was analyzed by transwell migration assay when these cells were incubated with the indicated conditioned medium for 48 h. **P < 0.01 ***P < 0.001

We examined the differentially expressed genes in CAFs under visfatin treatment using next- generation sequencing. As evidenced by KEGG analysis, JAK-STAT signaling pathway was found as one of the significantly activated pathways in CAFs upon the treatment of visfatin (Fig. 4A). Indeed, the phosphorylation levels of JAK2 and STAT3 were increased in response to visfatin treatment, whereas the phosphorylation levels of STAT5 remained unchanged (Fig. 4B). The JAK2-selective inhibitor, AG490, restored visfatin-induced upregulation of the phosphorylation levels of JAK2, as well as the expression of OCT4, Bmi-1, α-SMA and FSP-1 (Fig. 4C). In addition, the increased mRNA levels of inflammatory factors in visfatin-challenged CAFs were largely abolished by AG490 (Fig. 4D). Consistently, visfatin-treated CAFs CM-mediated migration of CRC cells was substantially abrogated when CAFs were treated with visfatin in the presence of AG490 (Fig. 4E). Together, these results suggest that visfatin activates JAK-STAT pathway in CAFs, which is required for visfatin-induced production of inflammatory factors in CAFs.

### Visfatin activates JAK2-STAT3 signaling in CAFs in a ROS dependent manner

**Fig. 5:**
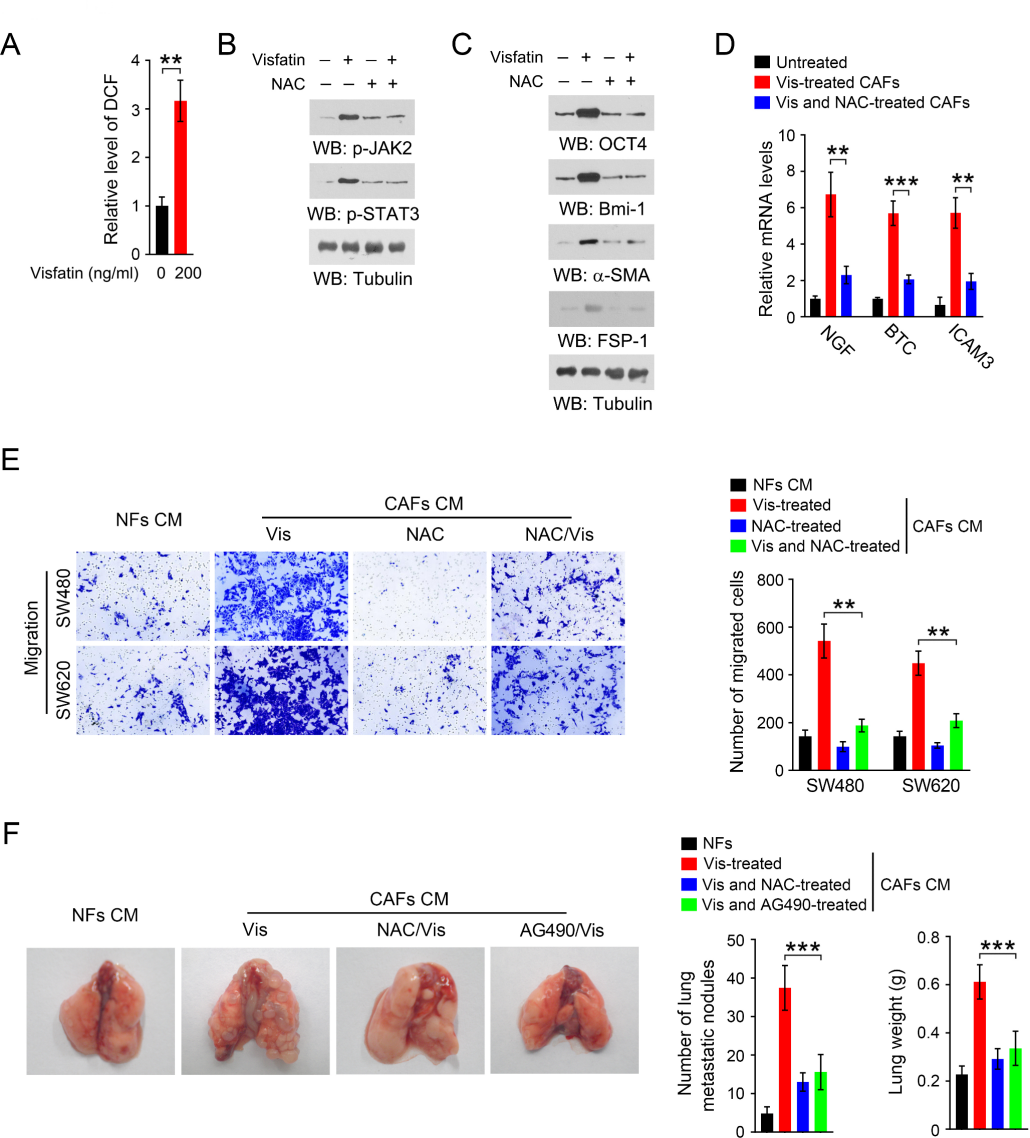
Visfatin induces ROS accumulation in CAFs. **A** CAFs were treated with Visfatin for 48 h, and the cellular ROS level was measured by using DCF probe. **P < 0.01. **B** CAFs were treated with Visfatin in the presence or absence of 10 mM NAC for 48 h and immunoblot analyses were performed using the indicated antibodies. **C** CAFs were treated with Visfatin in the presence or absence of 10 mM NAC for 48 h, and immunoblot analyses were performed using the indicated antibodies. **D** CAFs were treated with Visfatin in the presence or absence of 10 mM NAC for 48 h. The mRNA levels of NGF, BTC, ICAM3 was analyzed by RT-PCR. **P < 0.01 ***P < 0.001. **E** CAFs were treated with Visfatin in the presence or absence of 10 mM NAC for 48 h. The CAFs were cultured with fresh medium for another 48 h, and the conditioned medium was collected. The migration of SW480 and SW620 cells was analyzed by transwell migration assay when these cells were incubated with the indicated conditioned medium for 48 h. **P < 0.01. **F** SW480 cells were incubated with indicated conditioned medium for 48 h, and then the cells were injected into nude mice from the tail vein to establish the lung metastasis tumor model (n = 7). Representative images of mice lung were shown (left panel). The number of lung metastatic nodules and the lung weight was measured (right panel). ***P < 0.001

Oxidative stress is widely accepted to be involved in the activation and phenotypic remodeling of CAFs (Wu et al. 2021). Previous studies reported that visfatin could promote the production of ROS (Song et al. 2014). Thus, we asked whether ROS was involved in visfatin-mediated phenotypic remodeling of CAFs. As shown in Fig. 5A, visfatin treatment significantly increased ROS levels in CAFs. Treatment of the ROS scavenger N-acetylcysteine (NAC) markedly reduced the phosphorylation of JAK2 and STAT3 (Fig. 5B), protein expression of OCT4, Bmi-1, α-SMA and FSP-1 (Fig. 5C), as well as the mRNA levels of NGF, BTC and ICAM3 in CAFs (Fig. 5D). Moreover, visfatin-treated CAFs CM-mediated migration of CRC cells was largely abolished when CAFs were treated with visfatin in the presence of NAC (Fig. 5E). To further expand our findings, we verified the role of JAK/STAT pathway and ROS in visfatin-treated CAFs CM-induced CRC metastasis in mouse model. As expected, treatment with either AG490 or NAC substantially alleviated visfatin-treated CAFs CM-mediated CRC cell metastasis to mouse lung, shown by the reduced number of lung metastatic nodules and lung weight (Fig. 5F). Together, these results suggest that visfatin activates JAK2-STAT3 signaling in CAFs in a ROS dependent manner and oxidative stress and JAK2-STAT3 signaling are required for the pro-metastatic effect of visfatin-stimulated CAFs in CRC.

## Discussion

Tumor metastasis is a complex process that involves the metastasis of cancer cells from the primary tumor to distant organs or tissues (Mathivanan et al. 2010). Over the past decades, the focus of tumor metastasis studies has gradually transferred from cancer cells to the influence of the tumor microenvironment (Quan et al. 2019). The tumor microenvironment can directly or indirectly regulate cancer cells through cytokines or chemokines to promote the occurrence, development, and deterioration of tumors (Quail and Joyce 2013). CAFs, the main stromal cells in the tumor microenvironment, can be activated by a variety of cytokines to synthesize stromal components and secrete cytokines, both of which participate in tumor metastasis (Arneth 2019). Thus, exploring the role and mechanism of CAFs in CRC metastasis is of great importance to the prevention and treatment of CRC. In this study, we investigated the regulatory role of visfatin on CAFs and its effect on CAFs- derived factors and CRC metastasis. We found that visfatin activates JAK2-STAT3 signaling pathway and enhances ROS production, leading to the secretion of inflammatory factors to promote CRC metastasis.

CAFs are tumor stromal cells with diverse sources and heterogeneity (Erdogan and Webb 2017). Studies have shown that secreted proteins play important roles in the activation and phenotypic remodeling of CAFs (Tian et al. 2017). Extracellular visfatin, as a pro-inflammatory cytokine, has been widely involved in the development of CRC and other malignancies (Carbone et al. 2017; Wang et al. 2011). Previous studies have confirmed that visfatin can activate the TGF-β signaling pathway (Soncini et al. 2014). In addition, a variety of cytokines, such as VEGF and TGF-β, were conducive to the activation of CAFs (Ziani et al. 2021), suggesting that visfatin may play a role in the phenotypic remodeling and stemness of CAFs. In this report, we found that expression of a couple of inflammatory factors, including NGF, BTC and ICAM3 were increased in visfatin-stimulated CAFs. The upregulation of NGF in visfatin-stimulated CAFs was consistent with our previous study showing that high level of NGF was positively correlated with high incidence of CRC metastasis (Lei et al. 2022). In addition, it is reported that ICAM3 induced tumor metastasis through an LFA-1-ICAM3-ERM axis (Shen et al. 2018). BTC promoted ovarian cancer cell migration by enhancing Connexin 43 via MEK-ERK signaling (Zhao et al. 2020). Further work will be conducted to verify whether BTC and ICAM-3 also have a role in regulation CRC metastasis.

The activation status of JAK/STAT pathway, which plays a crucial role in the regulation of fibroblast phenotype, is susceptible to extracellular factors (Philips et al. 2022). The phosphorylated STAT3 has been reported to be acetylated by acetyltransferase to regulate tumor progression (Kuzet and Gaggioli 2016). Moreover, a previous study found that visfatin regulated the activity of the deacetylase Sirt1, pointing the potential link between visfatin and JAK/STAT pathway (Tran et al. 2016). In this study, to determine the regulatory mechanism of the stemness enhancement of CAFs by visfatin, we used high-throughput sequencing to analyze and enrich the differentially expressed genes in CAFs with or without visfatin treatment. Interestingly, JAK-STAT signaling was significantly enriched, and the activation of this pathway was then verified. Further, the JAK2-selective inhibitor AG490 counteracted the effect of visfatin on the inflammatory factors production in CAFs and CAFs-mediated migration and invasion of CRC cells. Therefore, our results suggest that JAK2 might a potential target to cut off the crosstalk between CRC cells and CAFs, which may benefit CRC treatment.

## Supplementary Information

Below is the link to the electronic supplementary material.

Supplementary file1 (DOCX 217 kb)

## Data Availability

The datasets used during the current study are available from the corresponding author on reasonable request.
