# Supplementary figures and images for: Extracellular nicotinamide phosphoribosyltransferase visfatin activates JAK2-STAT3 pathway in cancer-associated fibroblasts to promote colorectal cancer metastasis
Source: Genes Genomics. 2024 Dec 6;47(5):615–24. doi: 10.1007/s13258-024-01596-6 (PMC12081565; doi:10.1007/s13258-024-01596-6)

**Supplementary Figure 1**

**Unedited images of unedited immunoblots**


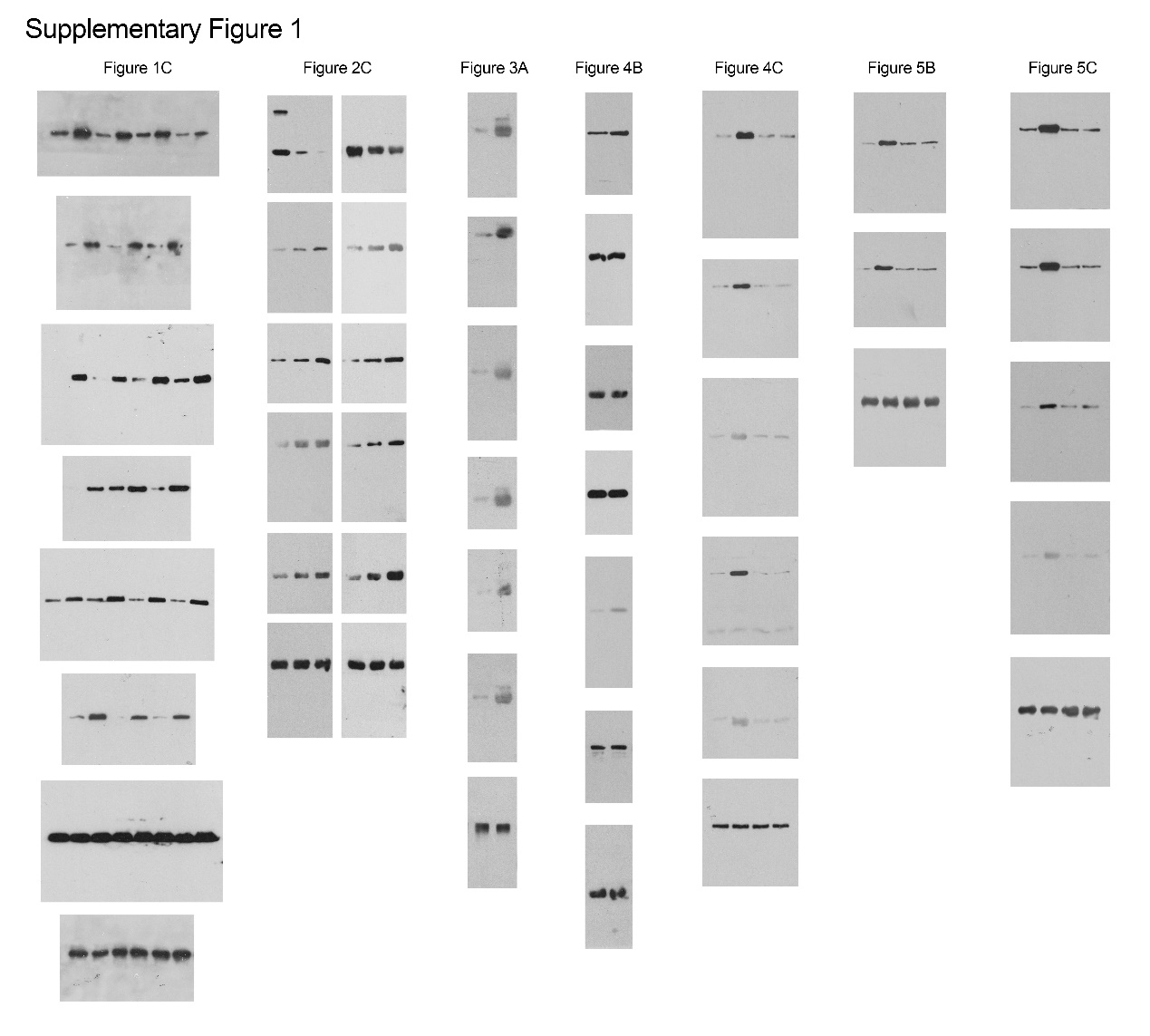

Supplement: Supplementary file 1 — Supplementary file1 (DOCX 217 kb) [file 13258_2024_1596_MOESM1_ESM.docx]
